# Elucidating interfacial failure of cervical restorations using damage mechanics: A finite element analysis

**DOI:** 10.1016/j.jds.2024.05.033

**Published:** 2024-06-12

**Authors:** Po-Chun Tseng, Shu-Fen Chuang, Ellen Schulz-Kornas, Karl-Heinz Kunzelmann, Andreas Kessler

**Affiliations:** a Department of Conservative Dentistry and Periodontology, University Hospital, LMU Munich, Munich, Germany; b School of Dentistry and Institute of Oral Medicine, College of Medicine, National Cheng Kung University, Tainan, Taiwan; c Department of Stomatology, National Cheng Kung University Hospital, Tainan, Taiwan; d Department of Cariology, Endodontology and Periodontology, University of Leipzig, Leipzig, Germany; e Department of Prosthetic Dentistry, Faculty of Medicine, Center for Dental Medicine, Medical Center-University of Freiburg, Freiburg, Germany

**Keywords:** Debonding, Dental restorations, Finite element analysis

## Abstract

**Background/purpose:**

Although clinical studies have suggested a link between non-axial forces and reduced longevity of cervical restorations, the underlying mechanisms require further numerical investigation. This in-silico study employed a cohesive zone model (CZM) to investigate interfacial damage in a cervical restoration subjected to different load directions.

**Materials and methods:**

A plane strain model of a maxillary premolar was established, with a wedge-shaped buccal cervical restoration. To simulate debonding, the restoration-tooth interface was modeled by the CZM, which defines the strain-softening damage behavior based on interfacial stress and fracture energy. Occlusal loads were applied in three different directions: (1) obliquely on the buccal triangular ridge, (2) obliquely on the palatal triangular ridge, and (3) equal magnitude axially on both ridges. Damage initiation and progression were analyzed, and stress distribution in damaged models was compared with the corresponding perfect-bond models.

**Results:**

Non-axial oblique loads initiated damage at lower forces (100 N for buccal and 120 N for palatal) compared to axial loads (130 N on both ridges). After debonding, buccal oblique loading caused higher stress at the central groove (42.5 MPa at 150 N). Furthermore, buccal oblique loading resulted in more extensive debonding than that caused by the palatal oblique load (88.3% vs. 43.3% of the bonding interface at 150 N).

**Conclusion:**

The study provides numerical evidence supporting the tooth flexure hypothesis, that non-axial forces are more detrimental to the bonding interface of the cervical restoration. The results highlight the necessity of damage mechanics in deriving stress distribution upon debonding.

## Introduction

In the absence of dental caries, chronic loss of cervical tooth tissue may still occur due to wear, erosion, and abfraction.^1^ The resulting lesions are often referred to as noncarious cervical lesions (NCCLs). A cross-sectional study has reported that 67.8% of patients admitted to a dental school clinic had at least one NCCL lesion.^2^ If left unrestored, the lesions are prone to progress with age, posing a threat to pulp vitality and structural integrity of the affected teeth.^1^

Despite the importance of cervical restorations in arresting NCCL progression, achieving long-lasting retention and interfacial integrity remains a challenge in clinical practice. Compromised bond integrity can lead to negative consequences, such as hypersensitivity and secondary caries.^3^ On average, 24% of the cervical restorations may exhibit marginal discoloration and 10% would be lost within 3 years.^4^

Occlusal stress has been recognized as a key contributing factor to NCCLs. Clinical studies have shown correlations between parafunctional habits, wear facets, and the presence of NCCLs, suggesting that excessive occlusal loads contribute to the development of these cervical defects.^5^^,^^6^ Likewise, cervical restorations are at a higher risk of debonding in teeth with wear facets.^3^ Since non-axial occlusal loads can be transferred through the crown and create concentrated stress in the cervical region of the tooth, the non-axial loads and the resulting tooth flexure are hypothesized to be a primary cause of interfacial failures observed around cervical restorations.^7^^,^^8^

Finite element analysis (FEA) has been utilized extensively in in-silico dental biomechanics to model clinically relevant scenarios.^9^, ^10^, ^11^ Despite the difficulty to model local variations in material properties, previous FEA still provided valuable insights into the impact of occlusal loads on cervical restorations, demonstrating that non-axial forces are associated with higher tensile stress at the bonding interface.^8^^,^^10^ However, a key limitation of conventional FEA models is the assumption of a perfect bond at the restoration-tooth interface. This assumption prevents them from capturing the process of bond deterioration and limits their validity to the point of interfacial damage initiation.

To overcome the limitations of the conventional FEA, damage mechanics must be incorporated to properly model interfacial debonding. In this study, we simulate the interfacial damage using a cohesive zone model (CZM) to derive the extent of damage and its consequences. Originally developed to simulate the crack for brittle fracture, the CZM has been successfully adapted to model debonding in adhesive joints, including the restoration-tooth interfaces.^12^^,^^13^ For comparison, we also build FEA models assuming perfect bonding at the interface. This allows us to directly assess the influence of interfacial damage on stress distribution.^14^

The null hypotheses tested in this study were: (1) The extent of interfacial damage would not differ due to variations in occlusal force direction. (2) The presence of interfacial damage had no effect on the maximum principal stress distribution.

## Materials and methods

### Geometry and mesh

**Figure 1:**
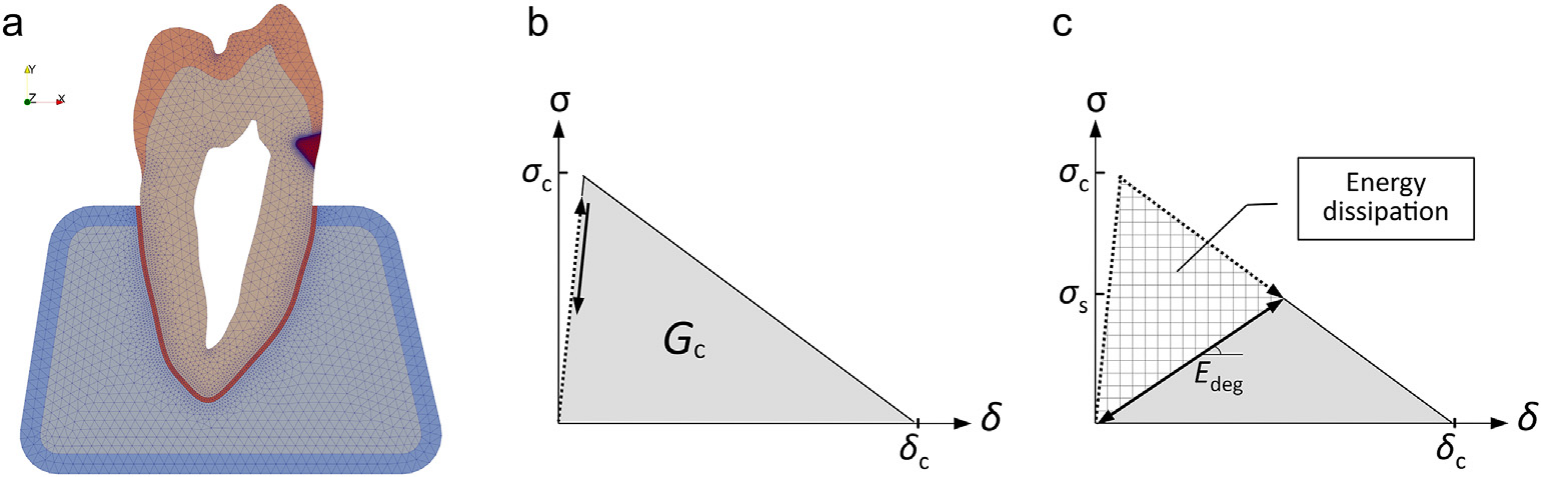
Meshed geometry and the strain-softening behavior defined by the cohesive zone model (CZM). (a) Meshed geometry of the tooth-PDL-bone structure with a simplified wedge-shaped restoration inserted at the buccal cervical region. (b) The CZM traction-separation curve defining the damage behavior of the interface by the interfacial stress (σ) and separation (δ). Critical stress (σ_c_) indicates the maximum permissible stress for the interface. (c) Upon reaching the critical stress, interfacial elements become damaged and demonstrate strain-softening behavior (with a reduced interfacial stiffness E_deg_). Once energy dissipation (hatched area) reaches the critical energy release rate G_c_, the element fractures and no longer bears the stress.

A 2D plane strain model of a maxillary human premolar was built based on a sagittal slice of a micro-CT scan.^15^ A wedge-shaped composite restoration for an artificial NCCL, consisting of an equilateral triangle with a 1.85 mm base and a 0.3 mm fillet at the apex, was incorporated at the buccal cervical region. The surrounding periodontal structures, including the periodontal ligament, cortical bone, and trabecular bone, were included to better represent the clinical scenario. The structures were meshed with first-order triangular elements using the Netgen algorithm (Fig. 1a). The global mesh size ranged between 0.25 and 0.0025 mm, with a finer mesh employed in critical areas: 0.1 mm for the periodontal ligament, 0.125 mm for the composite restoration, and a refined element size of 0.025 mm along the bonding interface.

Mesh convergence was evaluated with respect to the greatest maximum principal stress value and the damaged proportion of the bonding interface. By conducting computations on meshes with increasing density, convergence was ensured since both metrics converged within a 1% tolerance.

### Numerical simulation

The analysis was conducted using the open-source Salome-Meca suite (v2023 W64, EDF, Paris, France).^16^ In this study, the composite and the dental components were modeled as homogeneous and isotropically elastic materials. Except for the enamel-composite and dentin-composite interfaces, all interfacial connections between the tooth and supporting structures were modeled as perfectly bonded.

To simulate the debonding phenomenon at the restoration-tooth interface, a damage mechanics-based cohesive zone model (CZM) was employed. This CZM utilizes a bilinear traction-separation law, as illustrated in Fig. 1b. The bilinear CZM law was chosen to model the mixed-mode brittle fracture during debonding.^13^^,^^14^^,^^17^ When the interfacial elements experience stress exceeding a critical value, they exhibit strain softening damage, as the microcrack forming process observed during debonding Fig. 1c.^18^^,^^19^ The interfacial element completely debonds after dissipating the specified fracture energy. The input material and interface property values are listed in Table 1.

**Table 1:** Properties of the material and the interface.

Components	Poisson's ratio	Elastic modulus [MPa]
Enamel,^26^^,^^32^^,^^a^	0.30	84,100
Dentin^26^^,^^32^	0.30	18,600
Composite^33^	0.35	12,000
Periodontal ligament^10^	0.45	68.9
Cortical bone^10^	0.30	13,700
Trabecular bone^10^	0.30	1370
Interface	Critical stress [MPa]	Critical energy release rate [mJ/mm^2^]
Enamel-composite^34^^,^^35^	34.5	0.05
Dentin-composite^36^, ^37^, ^38^	17.0	0.01

a Based on the literature listed in references.

The finite element solver performed nonlinear quasi-static simulation using a semi-automatic timestepping algorithm. As a simplified approximation of masticatory strokes, nodal occlusal loads were applied incrementally from 0 N to 150 N in three distinct directions: (1) 45° oblique to the long axis on the buccal triangular ridge (incursive phase I), (2) 45° oblique to the long axis on the palatal triangular ridge (excursive phase II), and (3) two equal-magnitude axial forces applied on both the buccal and palatal triangular ridges (maximum intercuspation).^6^ The bottom of the cortical bone was constrained in all directions.

To illustrate the effect of debonding on stress distribution, additional FEA models were created analogous to the conventional perfect-bond model. In these models, the interface was assigned exceptionally high critical stress and initial stiffness values (1000 times the value used in the damage mechanics model), essentially preventing debonding throughout the simulation.

### Data analysis

During the post-processing stage of the simulation, the maximum principal stress was derived as an indicator of potential semi-brittle fracture initiation.^20^ Results were visualized using the Paravis module within the Salome-Meca suite. The extent of the damaged interface was extracted and then visualized using R (version 4.1.2) and the ggplot2 package (version 3.3.5). To assess the influence of debonding, the stress difference maps were generated by subtracting the stress distribution of the perfect-bond models from that of the corresponding CZM models. Finally, to facilitate further application of the damage mechanics in dental FEA, the data and code were deposited in an open-access online repository (https://doi.org/10.17605/OSF.IO/84ZGY).

## Results

### Damage initiation and progression

**Figure 2:**
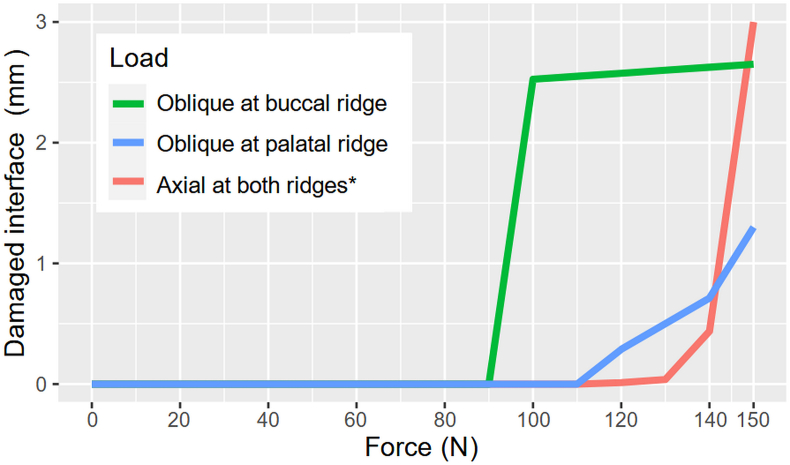
Line plot of damage progression under the three loading regimes, plotted against the corresponding magnitudes of the applied loads on the x-axis. For the axial loading case, two equal-magnitude forces were applied on both ridges, resulting in a total axial force twice the value indicated on the x-axis. Therefore, the line plots indicate that the bonding interface is more resistant to the axial loads.

An oblique load of only 100 N applied to the buccal ridge can induce extensive damage (Fig. 2, line plots illustrating the relationship between the applied load magnitude and the damage). In addition, palatal oblique loading results in less interfacial damage compared to buccal oblique loading. Table 2 further details the damage initiation force and the ultimate damage length at 150 N. The results indicate that the bonding interface is more resistant to axial loads compared to oblique loading scenarios.

**Table 2:** Force magnitude at damage initiation and the proportion of the damaged interface at 150 N.

Loading regime	Damage initiation force [N]	Damaged proportion at 150 N [%]
Buccal oblique	100	88.3
Palatal oblique	120	43.3
Axial at both ridges	130	100

∗For axial loading, two equal-magnitude loads were applied on both ridges. Therefore, the tooth received twice the axial force as indicated in the table.

### Damage distribution at 150 N

**Figure 3:**
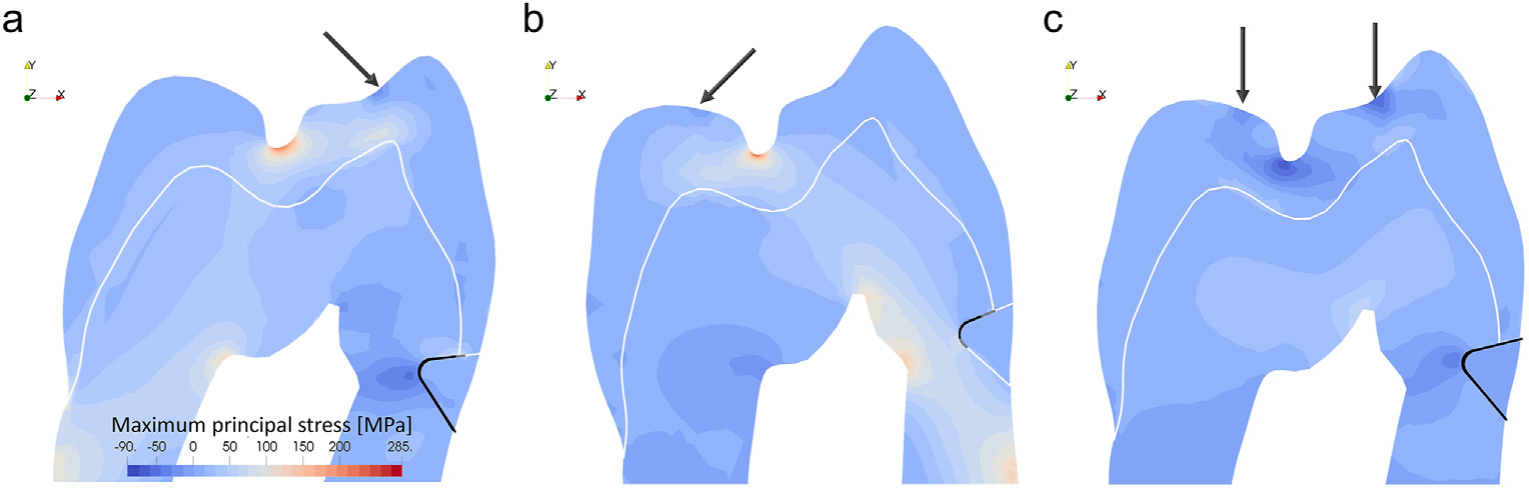
Maximum principal stress and interfacial damage at 150 N. Interfacial damage (black) was revealed in the deformed shapes (scale factor of 3) for better visualization. The arrows indicate the direction of the applied forces. (a) Under buccal loading, only a portion of the enamel-composite interface remains bonded. (b) In contrast, most of the interface was intact under the palatal load. (c) The interface was completely debonded when subjected to a combined axial load of 150 N on both ridges. However, it is important to note that the damage was not initiated until 130 N axial loads on both ridges.

Fig. 3 illustrates the spatial distribution of interfacial damage along with the corresponding maximum principal stress distribution for the three loading scenarios at 150 N. The results reveal extensive damage (88.3%) under buccal oblique loading, with complete debonding of the restoration-dentin interface and partial debonding of the restoration-enamel bond. In contrast, the palatal oblique load of 150 N resulted in more limited damage, affecting only 43.3% of the bonding interface near the cavity apex. Axial loading caused complete debonding at the maximum applied load (more specifically, 150 N on both buccal and palatal triangular ridges), but it is worth noting that the interface can well withstand axial loads up to 120 N on both ridges without any damage.

### Implications of damage

**Figure 4:**
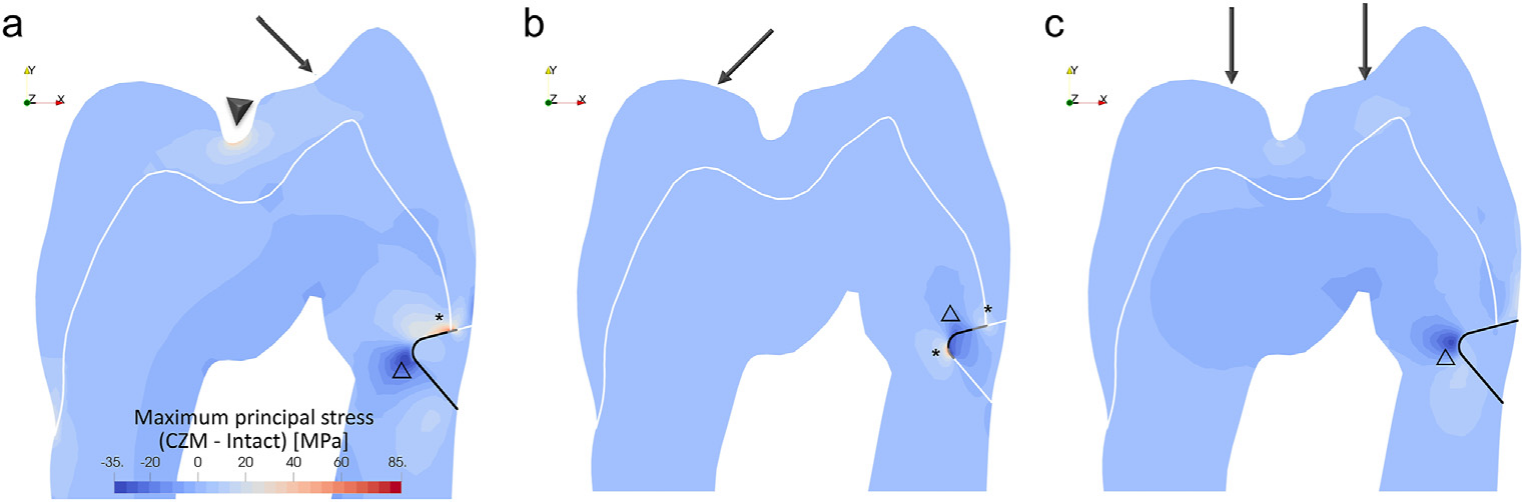
Difference maps of maximum principal stress distribution illustrating the effects of interfacial damage. Positive values indicate regions where the damaged models experience higher stress compared to the perfect-bond model under the same loading condition. The presence of damage leads to local stress relief (△) but also stress concentrations at the damage front (∗). Furthermore, the figure highlights a region of increased stress (▼) at the central groove in the buccally loaded damaged model.

Fig. 4 illustrates the difference in maximum principal stress between the CZM damage models and their corresponding perfect-bond models. Positive values indicate regions where the CZM models experience higher stress compared to the perfect-bond scenarios. The presence of interfacial damage, as indicated by the hollow triangle (△), leads to localized stress relief around the center of the damaged zones. However, it also induces stress concentrations at the advancing fronts of the damage (asterisks). Notably, under buccal loading, the damage model exhibits a particularly substantial increase in stress, reaching 42.5 MPa at the central groove (Fig. 4a, ▼). These observations indicate that interfacial damage can alter stress distribution at both local and distant areas within the restoration-tooth complex.

## Discussion

This study employed the CZM to investigate the complex, non-linear interfacial damage process around cervical restorations. The results demonstrate that the restoration-tooth interface exhibits greater resistance to axial loading compared to non-axial loading scenarios. These findings support the concept of tooth flexure as a key contributing factor to the debonding of cervical restorations, providing the first numerical evidence using a damage mechanics approach. Furthermore, the analysis revealed that interfacial damage alters the overall stress distribution within the restoration-tooth structure. This observation highlights the importance of incorporating damage mechanics approaches for a more comprehensive understanding of clinical scenarios involving debonding. Based on these results, the two null hypotheses are rejected, indicating that interfacial damage varies with occlusal force direction and alters stress distribution at the occlusal surface.

Considering the observed influence of interfacial damage on stress distribution, this study offers valuable insights for the management of NCCLs and provides further evidence for existing concepts. Before receiving cervical restorations, patients with occlusal wear facets or tooth malalignment should undergo a thorough evaluation of occlusal contacts, particularly during eccentric movements to identify premature contacts.^1^^,^^2^ Early restoration failure may suggest the need for re-examining occlusal contacts and reducing non-axial loading on the affected tooth. This highlights the importance of minimizing excessive non-axial forces, also a well-recognized risk factor for NCCLs.^2^ Patients with parafunctional habits, such as bruxism, might require additional considerations. In certain scenarios, occlusal adjustments or the use of occlusal splints could be beneficial. The study also found a correlation between interfacial damage and increased stress at the occlusal groove, amplifying the existing stress concentration at the grooves of intact teeth during mastication.^9^ This may elevate the risk of tooth fracture, similar to what is observed in cases of unrestored cervical defects.^21^^,^^22^ By incorporating interfacial damage mechanics into the FEA framework, this study represents a significant step toward achieving more clinically relevant simulations.

While clinical studies offer the most applicable information for clinical practice, their results are usually confounded by various clinician-, patient-, and even defect-dependent factors. Therefore, it is challenging to obtain mechanistic insights directly from the clinical studies. On the other hand, laboratory studies provide a well-controlled approach to isolate the effects of specific variables. However, since each tooth is unique and can only be tested once, researchers can only strive to standardize experimental procedures as much as possible. Accordingly, a complex interplay among variations and other uncertainties is inevitable, posing a challenge to dissect the contribution of each variable under investigation.

FEA offers a valuable tool to illustrate mechanical phenomena. By leveraging established physical principles, FEA can resolve the uncertainties introduced by anatomical variations and technical differences. Even though FEA can systematically delineate the effects of different variables, conventional FEA models often rely on the assumption of a perfect bond between the restoration and the tooth. This assumption limits their ability to model and predict the process of clinical bond deterioration, a crucial factor in the longevity of restorations. Furthermore, FEA based on the perfect bond assumption may derive unrealistic stress at the interface under physiological loading.

To address the limitation of the perfect-bond models, various techniques have been explored to approach debonding phenomena. The element deletion method, where interfacial connectivity is severed upon exceeding a predefined maximum stress threshold, constitutes the earliest attempt. However, this approach suffers from mesh dependency as it gives no consideration for energy dissipation.^23^ Alternatively, linear elastic fracture mechanics (LEFM) has also been employed to simulate crack propagation.^24^^,^^25^ However, this method requires computationally expensive step-wise remeshing, and it is not specifically designed for interfacial debonding scenarios.^13^ Another approach involves introducing predefined non-bonded interfaces within the model.^11^^,^^26^ The static approach avoids the complexities of simulating damage progression but cannot capture the progressive nature of debonding, potentially leading to significant deviations from real-world clinical situations.^14^ These limitations highlight the need for more rigorous models that can incorporate damage mechanics and realistically represent the initiation and propagation of interfacial debonding.

By introducing the CZM into dental FEA, our study opens a new avenue to rigorously model interfacial debonding, a crucial yet under-investigated clinical phenomenon. Unlike conventional static FEA, the CZM leverages damage mechanics principles to model the progression of debonding. This approach can also solve the intractable mesh dependency issue associated with the element deletion method. Furthermore, the CZM offers an additional advantage in terms of computational efficiency by eliminating the need for resource-intensive remeshing, which is required by the LEFM approach.

The damage distribution patterns in this study correlate well with in-vitro and clinical observations reported in the literature, highlighting the clinical relevance of our results. Nowadays, internal defects at the bonding interface can be revealed using optical coherence tomography (OCT),^27^ allowing non-invasive imaging for damage monitoring.^28^ After clinical service of 36–48 months, OCT has revealed a high proportion of defective restoration margin, with median values ranging from 47.9% to 92.8% depending on adhesive strategies employed.^29^ Besides, a clear link has been established between increased interfacial defect and retention loss.^30^ Thus, these findings strongly support the necessity of incorporating damage mechanics approaches into in-silico simulations.

The present study demonstrated the feasibility and value of CZM in modeling interfacial damage around NCCLs. As the first proof of concept, there are still aspects to be addressed to further refine and validate the novel approach. First, the model was built according to the plane strain condition, limiting the results to the mid-sagittal section. Extending the model into 3D would provide a more comprehensive picture of damage and stress distribution within the restoration-tooth complex. Second, further investigations are warranted to elucidate the extent of interfacial damage under polymerization shrinkage.^31^ Incorporating variations in relevant parameters such as the mechanical properties of restorative materials and cavity configurations would provide further insights for clinical guidance. Third, in-vitro experiments would be highly valuable to validate the model's predictions. Such experiments will need to consider anatomical variations and other potential uncertainties, but it would be advantageous to include material inhomogeneity for more accurate results via the use of realistic samples. Finally, the load directions could be systematically varied in future studies to further illustrate the relationship between load directions and interfacial damage.

In conclusion, this study represents a significant step towards achieving more clinically relevant FEA through the application of damage mechanics. The in-silico analysis provides numerical evidence for the tooth flexure hypothesis, demonstrating that non-axial forces are more detrimental to the cervical restoration's bonding interface. The analysis also reveals a crucial link between extensive interfacial damage and increased stress at the occlusal groove, putting the integrity of the tooth structure at a higher risk. To minimize detrimental debonding and ensure the longevity of cervical restorations, careful occlusal evaluation and proper management of non-axial occlusal forces are recommended before restoring NCCLs.

## Declaration of Generative AI and AI-assisted technologies in the writing process

Statement: During the preparation of this work the authors used Gemini, an artificial intelligence (AI) language model developed by Google, to improve the language of this manuscript. After using this service, the authors reviewed and edited the content as needed and take full responsibility for the content of the publication.

## Declaration of competing interest

The authors have no conflicts of interest relevant to this article.
